# An examination of handwritten signatures forged using photosensitive signature stamp

**DOI:** 10.1080/20961790.2021.1898755

**Published:** 2021-05-03

**Authors:** Zhen Li, Xinlai Liu

**Affiliations:** Department of Documents Examination, Criminal Investigation Police University of China, Shenyang, China

**Keywords:** Forensic sciences, questioned document, forensic document examination, signature examination, photosensitive stamp, microspectrophotometry, IR analysis, fluorescence analysis

## Abstract

Signature examination is the most common examination performed by any document examiner. Determination of the authenticity of a handwritten signature on a questioned document is an important task for forensic document examiners in the forensic science field. As a result of continuous developments in technology, a signature stamp can now be created using a photosensitive seal to enable the reproduction of a handwritten signature. These stamps are commonly used in China and several other countries. In this study, 10 types of black photosensitive stamp-pad ink, 10 brands of fountain pen ink, 15 types of black gel ink and six types of black erasable gel ink found on the Chinese domestic market were collected and 10 photosensitive signature stamps were created using the signatures of 10 people. Microscopic analysis, infrared (IR) and fluorescence analyses and microspectrophotometry (MSP) techniques were used to examine the resulting photosensitive signature stamp impressions when applied to printing papers, writing papers and invoice papers. By comparing the printing and spectral characteristics of the photosensitive signature stamp impressions with those of the signatures executed using the fountain pens, gel pens and erasable gel pens, it was possible to determine whether each signature was written or stamped using a photosensitive signature stamp. To validate these results, a 96.7% absolute accuracy and a 99.3% detection rate were achieved over a total of 150 blind tests conducted by five forensic document examiners, thus demonstrating that a combination of the four analysis methods used in this work can provide a more scientific approach and improve the accuracy and the detection rate of the examination process.

**KEY POINTS**
- A signature stamp is a photosensitive seal made in the style of a handwritten signature.
- Although microscopic analysis can usually provide better examination results, a comprehensive examination method that includes microscopic analysis and ink composition analysis is required to improve the accuracy and the detection rate of the examination process.
- This study collected and tested photosensitive stamp-pad inks, fountain pen inks, gel inks and erasable inks.
- Infrared and fluorescence analyses and microspectrophotometry were able to distinguish the photosensitive ink from both erasable ink and fountain pen ink.

Supplemental data for this article are available online at https://doi.org/10.1080/20961790.2021.1898755.

## Introduction

The examination of signatures is the most common task for most document examiners. Determination of the authenticity of a handwritten signature on a questioned document is a critical aspect of forensic document examinations and the first step is to determine whether the signature was created using writing ink. Identification of a signature or proving that it is genuine has been a prominent aspect of many legal controversies. Many studies have been performed on signature analysis in recent years and an extensive range of literature is available with regard to signature examination [1–12].

The legal definition of a signature is found in *Black’s Law Dictionary*: the act of putting one’s name at the end of an instrument to attest its validity; the name thus written [13]. A signature may be written by hand, printed, stamped, typewritten, photographed or cut from one document and then attached to another. Signatures on wills, deeds, notes, contracts and cheques are forged more frequently than the signatures to other documents, but signature forgery can occur in various classes of documents. As a result of technological changes, new methods to forge signatures are commonly being used. Disputes arise over the reproductions of signatures produced by colour photocopying, inkjet printing, and rubber stamps.

**Figure 1.**
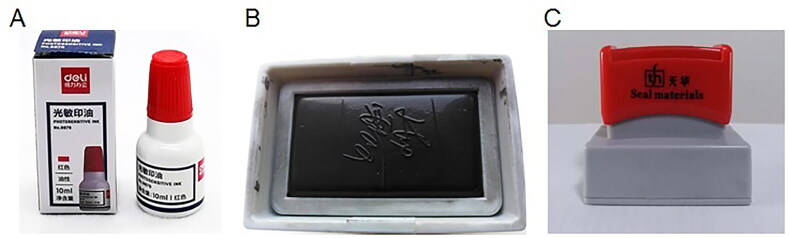
(A) Photosensitive stamp-pad ink stored in a bottle. (B) Surface of a photosensitive signature stamp. (C) Complete photosensitive signature stamp.

**Figure 2.**
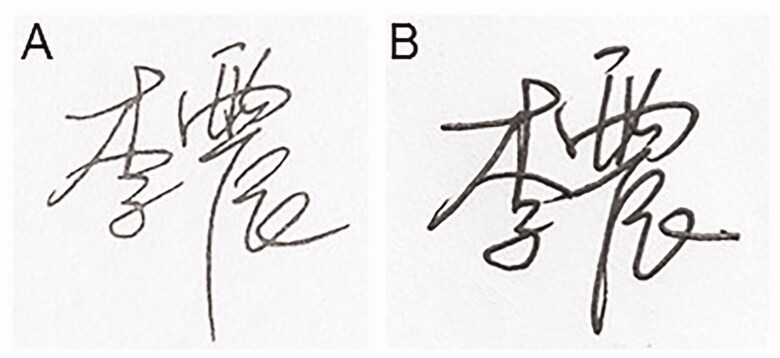
Photosensitive signature stamp impression (A) and handwritten signature (B).

In China, both signatures and stamps are applied frequently because of the country’s unique history and culture. These signatures and stamps are often affixed to official and private documents, including contracts, passports, cheques, receipts and other important documents. Many cases in China involve the examination of signatures, which represents a specialized branch of handwriting examination [14–23]. Traditionally, there is a history of use of name seals in China. The name seal, as it implies, is the printed typeface of the bearer’s name engraved on the seal’s surface. In recent years, the signature stamp, which is different to the name seal, has appeared in China. A signature stamp is a photosensitive seal fabricated in the style of a handwritten signature. Sometimes, the photosensitive signature stamp impression produced by such a stamp can appear to be a genuine handwritten signature, particularly to lay people. Images of a photosensitive signature stamp and a comparison of a signature stamp impression with a handwritten signature are shown in Figures 1 and 2, respectively. In some instances, even experienced document examiners may have difficulty in reaching a defini­tive conclusion about the signature’s authenticity in such cases.

To fabricate a photosensitive signature stamp, a handwritten signature must first be scanned into a computer *via* an external input device. Image processing software can then be used to edit and adjust the signature image where necessary. The edited signature stamp pattern is then printed onto tracing paper or a transparent film using a monochromatic laser. The positive-working plate of the signature printed on the tracing paper or transparent film is then placed on the photosensitive stamp pad and they are combined to create a photographic exposure. After this exposure, the non-signature portion of the image forms a black film with a specific thickness and strength that acts to block the micropores and ensure that the oil cannot penetrate it. The photosensitive material in the signature part has not experienced photosensitivity and thus remains in its original state. Finally, the exposed photosensitive pad is filled with a special ink. During the stamping process, the ink in the pad will seep through the micropores to form a stamp impression of the signature.

However, no papers related to signature stamps have been retrieved from the literature to date. The research on signature stamps is still lacking. Forensic examinations of questioned documents routinely involve physical and chemical analyses of the inks used. The ink composition is determined *via* optical inspection, spectroscopy, chromatography, mass spectrometry, dissolution and other methods. Among the above methods, chromatography, dissolution methods and mass spectrometry are destructive and cannot maintain the integrity of the samples, which affects any further examination of the inks. Spectroscopic methods have been widely used in examinations of inks, taking advantage of the fast and nondestructive nature of these methods and the fact that sample pretreatment is not required.

Braz et al. [24] analysed the handwriting produced on documents using different pens *via* Raman spectroscopy. They found that Raman spectroscopy can be used to distinguish the different types of ink used. Zięba-Palus and Kunicki [25] examined approximately 70 blue and black ballpoint and gel pens using techniques including micro-Fourier transform IR (FTIR) and Raman spectroscopies and X-ray fluorescence (XRF) analysis. The ink composition can be analysed using all three of these methods. It is also possible to differentiate between inks of the same colour. Based on the infrared and Raman spectra obtained, approximately 95% of blue and black inks can be distinguished. In the case of the gel inks, up to 90% of the samples could be differentiated, depending on the colour of the gel ink.

Liu and Li [26] classified 12 erasable pen samples *via* IR spectroscopy, FTIR spectroscopy, fluorescence analysis and microspectrophotometry. Their results showed that different brands of erasable pens could be distinguished to some extent by their infrared spectra, FTIR spectra and fluorescence analysis. Microspectrophotometry proved more suitable for distinguishing blue inks when compared with black inks of different brands.

Reed et al. [27] analysed a variety of blue, red and black gel inks on white office paper using the hyperspectral imaging (HSI) method. HSI enables the detection of subtle differences between chemically similar inks. The potential of the HSI technique for ink discrimination was highlighted when compared with other analytical examination methods.

**Figure 3.**
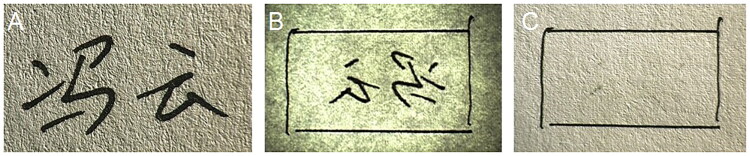
(A) Handwritten signature. (B) Handwritten signature under transmitted light conditions. (C) Handwritten signature showing no obvious writing indentations on the back of the paper. Simulation experiments show that in some cases, it is very difficult to determine whether or not a questioned signature is handwritten using microscopic analysis alone.

When determining whether a questioned signature was handwritten or is an impression made by a photosensitive signature stamp, it is necessary to use a comprehensive examination method that includes microscopic analysis and ink composition analysis. The authors previously participated in the examination of two questioned signatures. The first questioned signatures had writing indentation in a few strokes and showed none of the obvious stamping characteristics of a photosensitive signature stamp, which caused four forensic document exami­ners (FDEs) to give different opinions. The results of the simulation experiment are shown in Figure 3. In the examination of the other questioned signature, the suspect confessed that he had traced the strokes using an ink-free gel pen after stamping with a signature stamp, which brought unprecedented challenges to the signature examination process. This experience demonstrated that although microscopic analysis can usually provide better examination results, a comprehensive examination method that includes both microscopic analysis and ink composition analysis is required to improve the accuracy and the detection rate of the examination process. Ink composition analysis can determine the similarities and differences between the ink composition of the questioned signature and that of a specimen signature. In the face of complex cases of the type described above, when it is impossible to determine whether or not the questioned signature is handwritten using microscopic analysis, it remains possible to rule out the possibility that the questioned signature is an impression made using a photosensitive signature stamp by analysing the ink composition of the questioned signature. Therefore, a comprehensive examination method that includes microscopic analysis and ink composition analysis will help examiners to draw an accurate and reliable conclusion.

## Materials and methods

### Papers

Three types of paper were used in this study, including printing paper with a basis weight of 70 g/m^2^ (Yalong Paper Products Co., Ltd., Kunshan, China), writing paper with a basis weight of 40 g/m^2^ (Junrong Printing Technology Co., Ltd., Shanghai, China) and invoice paper with a basis weight of 20 g/m^2^ (Li Xi Paper Co., Ltd., Guangzhou, China).

### Stamp-pad inks and pens

A variety of writing and printing materials were used to prepare the sample signatures, including black photosensitive stamp-pad inks, gel inks, fountain pen inks and erasable gel inks:

10 types of photosensitive stamp-pad ink, 15 types of gel ink, 10 brands of fountain pen ink and six types of erasable gel ink are listed in Supplementary Tables S1–S4.

**Table 1.** Results of independent-sample *T* testing of ink GM-10.

Wavelength (nm)	Levene’s test for equality of variances			*T* test for equality of means			
	F	P*	*t*	P*	Difference		
					Mean	SE	95%CI
726.14	0.276	0.603	–0.236	0.815	–0.0216	0.0917	(–0.2091, 0.1659)
756.74	0.004	0.947	1.372	0.181	0.0513	0.0374	(–0.0250, 0.1279)
795.37	1.490	0.232	0.212	0.834	0.0114	0.0538	(–0.0990, 0.1215)
852.20	1.788	0.192	0.308	0.760	0.0150	0.0487	(–0.0847, 0.1147)
900.55	0.439	0.513	–0.054	0.958	–0.0029	0.0546	(–0.1146, 0.1087)

*P < 0.05 indicates significant difference.

### Preparation of samples

Ten laypersons participated in the preparation of the signature samples. Each person wrote their own signature every 60 s at their habitual speed and using normal pressure on the printing papers. No directions were given to these signature providers as to how to sign their signatures. In this experiment, 15 types of black gel pen, 10 brands of black fountain pen and six types of erasable gel pen were used, giving a total of 31 types of pens. Each person wrote five signatures with each type of pen to provide a total of 155 personal signatures. A signature written using a black gel pen was randomly selected from each person to be used to produce a photosensitive signature stamp. The 155 handwritten signatures created by each participant for this study gave a total of 1 550 prepared samples.

The second set of samples comprised 90 impressions that were stamped using the 10 photosensitive signature stamps described above. Three impressions were stamped with each photosensitive signature stamp on each type of paper. These impressions were stamped on the three papers using different brands of stamp-pad inks. All samples were stamped by the same person with no other specific requirements (i.e. following their own personal habits, rather than any particular instruction to increase or reduce the pressure used).

The third set of samples was prepared in a flowing manner. There were several sample grids on the three papers above in which the numbering corresponded to each sample. Ink spots from stamp-pad inks and fountain pen inks were deposited onto each sample grid or the letter “A” from gel inks and erasable gel inks were filled in sample grids. There were either two ink spots or one letter “A” in each sample grid. To maintain relative consistency of writing pressure, each letter “A” was written by the same person at their habitual speed and normal pressure on the three types of paper. In this way, a total of 183 samples were prepared. All samples were stored under controlled conditions at approximately room temperature (25 °C) with a relative humidity of 50% RH. The samples were stored individually in a room without being placed in folders or document pouches or being placed in direct contact with other documents.

### Apparatus

The following equipment was used in this study:

The Video Spectral Comparator 6000 (VSC6000; Foster & Freeman Ltd., Evesham, UK), including a charge-coupled device (CCD) camera, an infrared light source, a fluorescent light source, an optical filter and a high-resolution grating photometer.

Microspectrophotometer (MSP): Full Spectrum Microspectrophotometer (CRAIC AXIO, Madison, WI, USA), where the illumination system is the ZEISS N XBO 75 Microscope Illuminating System.

SteREO Discovery: V20 stereomicroscope (Carl Zeiss Co., Oberkochen, Germany) equipped with a ZEN Blue Lite soft.

### Experimental procedure

First, the microscopic examinations were performed using the SteREO Discovery. All impressions stamped using the 10 photosensitive signature stamps and the corresponding handwritten signatures were examined microscopically. The results of these exami­nations were recorded as image recordings.

Second, the third set of samples was placed on the sample stage of the VSC6000. The fluorescence and infrared spectroscopic systems were started and the wavelengths of each band were selected and used to irradiate the inks. The results of these examinations were again recorded as image recordings.

Finally, the third set of samples was placed on the sample stage of the MSP. The imaging system was started and the samples were placed at the centre of the xenon light spot. The light wavelength range of 400–1 000 nm was selected for the spectral measurements. The results of the examinations were also recorded visually *via* imaging. Infrared and fluorescence analyses and MSP were used to test the third set of samples. These examination methods include both physical and optical techniques.

## Results and discussion

### Observable effects of microscopic analysis

**Figure 4.**
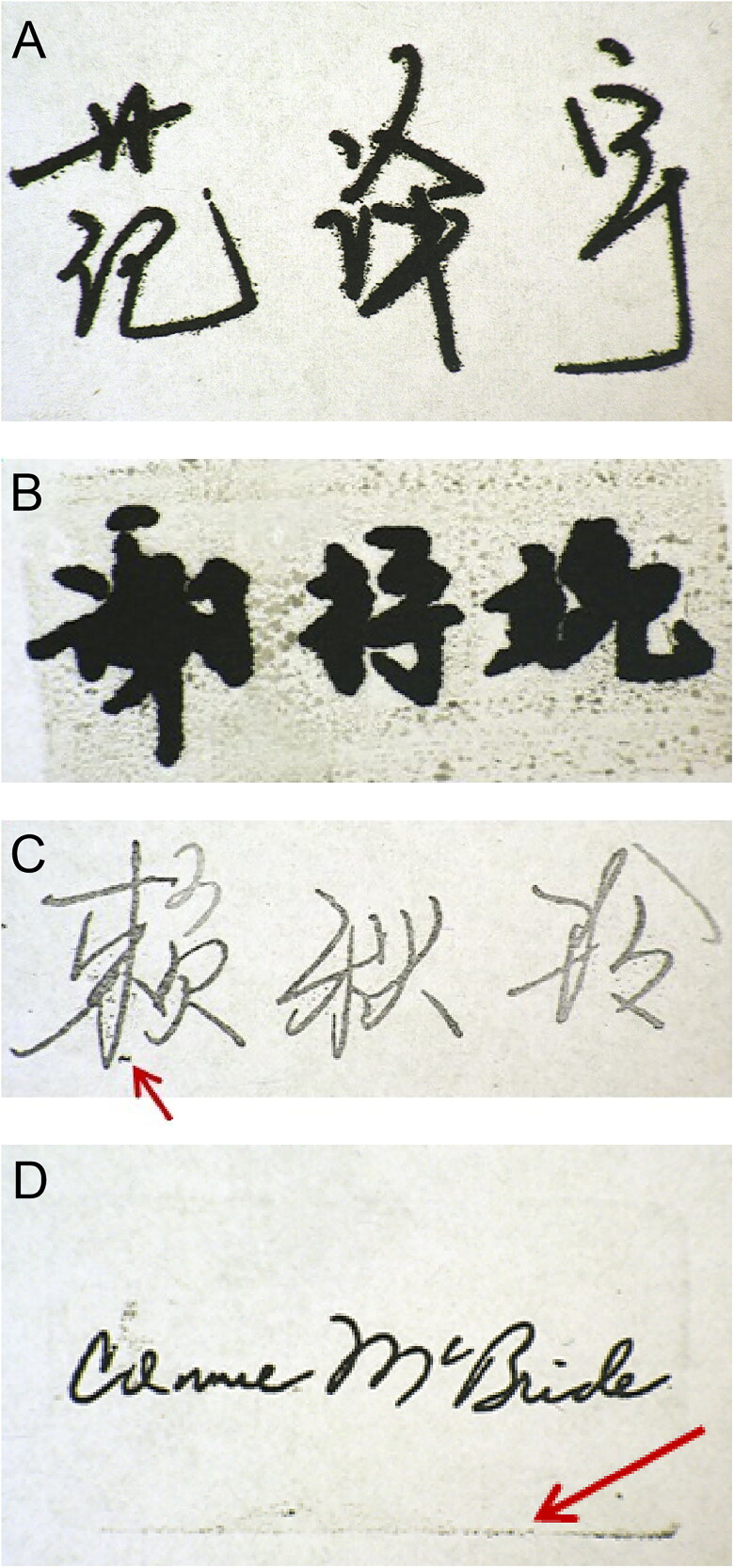
The strokes in a photosensitive signature stamp impression are usually not smooth, burr-shaped, or may even show a “squeegee effect” (A). Scattered ink (B) and ink defects (C) in blank areas are distinctive and unique features of photosensitive signature stamp impressions. Border feature of the stamp surface that commonly appears in photosensitive signature stamp impressions (D).

When compared with the corresponding handwritten signatures, the strokes in the signature stamp impressions were usually not smooth, were burr-shaped, or even exhibited a “squeegee effect”. A typical image is shown in Figure 4A. The signature stamp was also likely to be damaged, worn or contained void areas formed during production and use. When void areas, defects, nicks and cuts, edge wear and breakdown within the strokes were observed, this could be used as a basis for identification as a signature stamp impression in each case.

As shown in Figure 4B, a signature stamp impression could be distinguished from a handwritten signature by a distinctive and unique feature, which was the phenomenon of scattered ink appearing in blank areas with a high occurrence rate, particularly when the stamping force used was more intense or the stamp showed significant wear. If these ink defects appeared in the blank areas, they could also be included in the examination. The appearance of ink defects in blank areas is illustrated in Figure 4C.

The border features of the stamp surface were also sometimes reflected in the impression on the paper. Although these features had a low probability of occurrence, they would appear under specific circumstances. There were two main types of chara­cteristics: one was the ink frame trace, in which a coloured frame outline was left around the signature, and the other was frame indentation on the paper, which was a colourless paper surface deformation that could be observed using a sidelight. These features also reflected the outline of the stamp surface. A typical image of such a feature is shown in Figure 4D.

**Figure 5.**
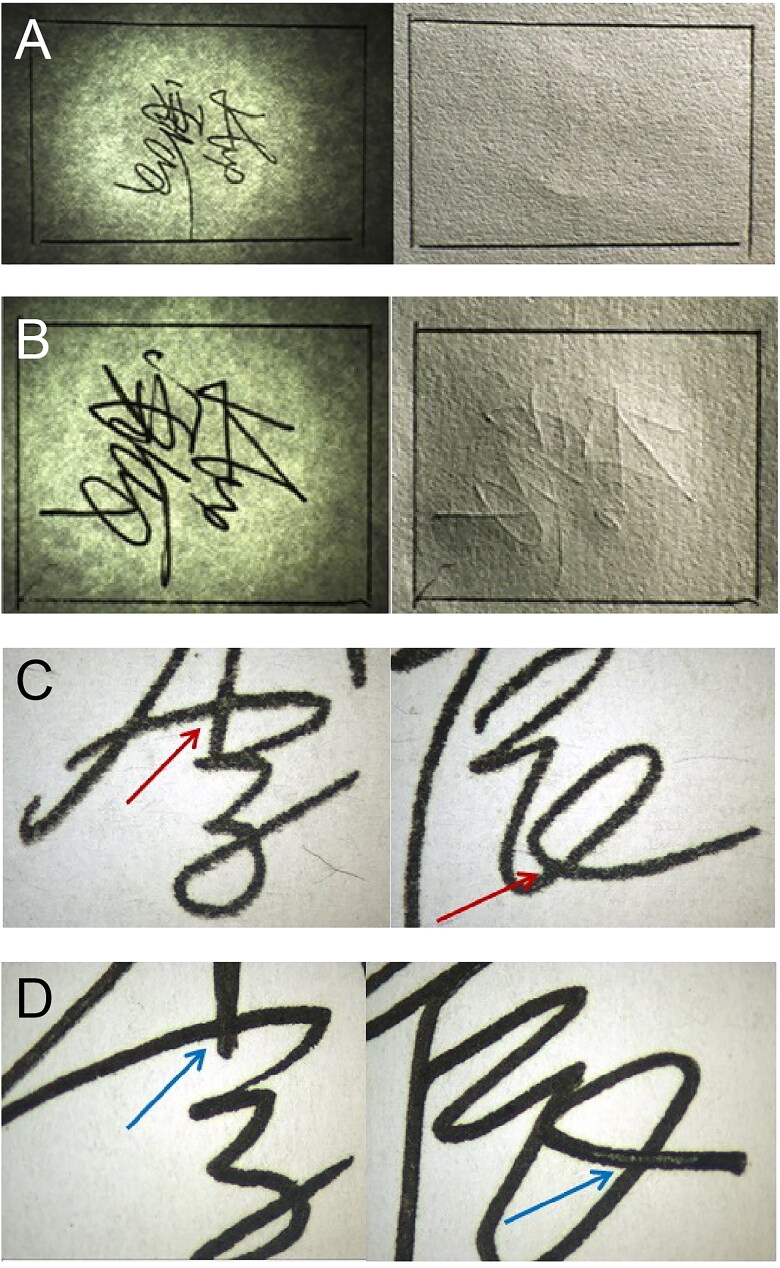
Photosensitive signature stamp impressions ­ge­nerally show no obvious indentations (A) when compared with a handwritten signature with obvious writing indentations on the back of the writing paper (B). The strokes in the photosensitive signature stamp impression (C) were flat and lacked depressions with no obvious three-dimensional effects at the crossing strokes, compared with the handwritten signature (D).

The writing formed using a signature stamp gene­rally showed no obvious indentation. The printed part of the photosensitive stamp was almost in the same plane as the blank part. During stamping, because of the large force area and the flexibility of the stamp surface material, the applied force was relatively uniform. Even if the pressure was increased, no indentation would be made on the paper. A typical image is shown in Figure 5A, B. As a result of the influence of photosensitive stamp-pad inks, some obvious traces of ink penetration were observed. When a signature was handwritten on paper, obvious writing indentations would show on the back of the writing paper and there would be almost no trace of ink penetration. The writing pressure and the paper thickness were the main factors that determined whether the indentation was obvious or not. Sometimes, when the writing pressure was lighter and the paper was thick, it was difficult to see any indentation. Therefore, it was not possible to determine that the signature was a signature stamp impression by the presence of indentation and oil penetration alone. However, as long as a writing indentation was present, it could be determined that the signature was handwritten.

The photosensitive stamp impressions showed clear writing and uniform colour distribution. In particular, there were no obvious three-dimensional effects at the crossing strokes, and it was also found that there were no light and dark changes under sidelight observation. In addition, under the V20 stereomicroscope, it was found that the strokes were flat and without depressions. When writing a signature normally, because of the uneven strength applied, the ink distribution on the paper is different, the colours of the strokes are light and uneven, and these factors have a three-dimensional effect. The edges of the strokes are neat and there is no evidence of the dense squeezing phenomenon that is often found in photosensitive signature stamp impressions. Typical images of these impressions are shown in Figure 5C, D. The characteristics of the signatures written using the different writing tools were also different. For example, when a gel pen (including an erasable gel pen) was used to write a signature, the strokes were more uniform. These strokes had a small amount of gloss and the edges of the strokes were also slightly blurred. Sometimes, more ink was present on the sides of the strokes and less ink was present in the middle. When a fountain pen was used to write the signature, the strokes were conti­nuous and smooth, the shading of the strokes was uneven and the edges of the strokes were rough because of ink penetration; these uneven jagged shapes can be observed under the microscope.

### Observable effects of infrared analysis

As irradiated in the Experimental procedure section, 10 types of black photosensitive stamp-pad ink, 15 types of black gel ink, 10 brands of black fountain pen ink and six types of black erasable gel ink on printing paper were irradiated under the VSC6000’s built-in infrared illumination conditions using continuous interference band-pass filters. Note of the 10 types of black photosensitive stamp-pad inks on printing paper were not changed under the infrared illumination conditions at 645, 695, 715, 850 or 1 000 nm (Supplement Figure S1).

**Figure 6.**
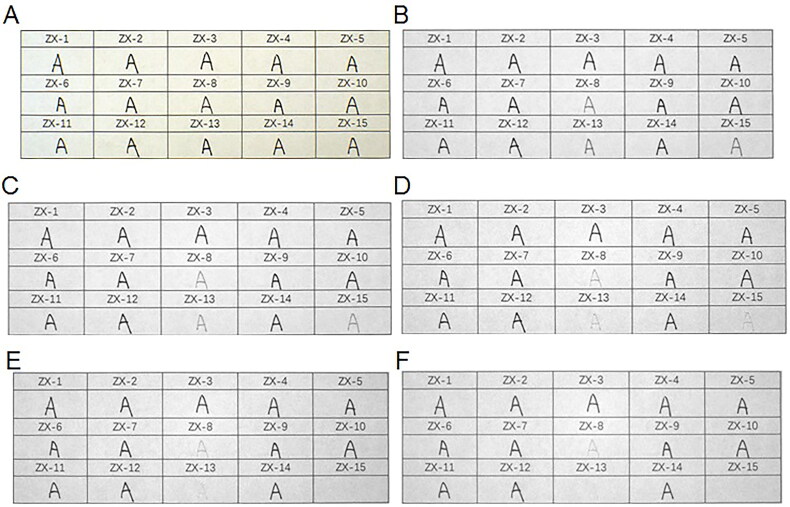
Changes in 15 types of black gel ink on printing paper obtained under infrared (IR) illumination conditions using continuous interference band-pass filters. (A) Original diagram under visible light. (B–F) IR absorption diagram at 645, 695, 715, 850, and 1 000 nm.

When the 15 types of black gel ink on printing paper were irradiated at 645 nm, the ZX-8, ZX-13 and ZX-15 inks started to lighten in colour but the other samples remained unchanged. The three types of inks were close to disappearing and disappeared completely when the irradiation wavelength was 715 and 1 000 nm, respectively (Figure 6).

**Figure 7.**
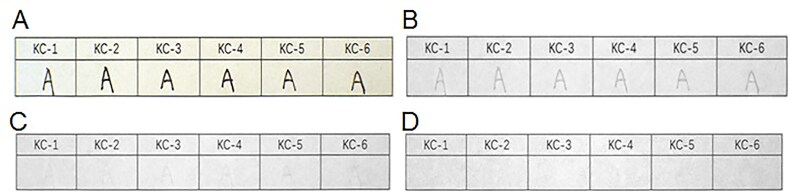
Changes in six types of erasable gel ink on printing paper obtained under infrared (IR) illumination conditions using continuous interference band-pass filters. (A) Original diagram under visible light. (B–D) IR absorption diagram at 645, 695 and 715 nm.

All the six types of black erasable gel ink on printing paper started to lighten in colour after irradiation at 645 nm, were close to disappearing after irradiation at 695 nm, and disappeared completely after irradiation at 715 nm (Figure 7).

**Figure 8.**
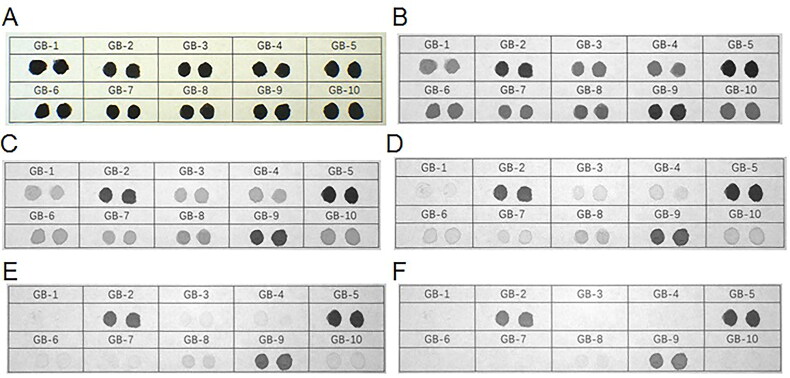
Changes in 10 types of black fountain pen ink on printing paper obtained under infrared (IR) illumination conditions using continuous interference band-pass filters. (A) Original diagram under visible light. (B–F) IR absorption diagram at 645, 695, 715, 850, and 1 000 nm.

The 10 brands of black fountain pen ink on printing paper started to lighten in colour after irradiation at 645 nm. When irradiated at 695 nm, inks GB-1, GB-3, GB-4, GB-6, GB-7, GB-8 and GB-10 started to lighten in colour. When irradiated at 715 nm, inks GB-3, GB-4, GB-6, GB-7, GB-8 and GB-10 continued to lighten, while ink GB-1 was close to disappearing. After irradiation at 850 nm, inks GB-3, GB-4, GB-6, GB-7, GB-8 and GB-10 were all close to disappearing and ink GB-1 disappeared completely. When the irradiation wavelength was 1 000 nm, inks GB-3, GB-4, GB-6, GB-7, GB-8 and GB-10 disappeared completely, and inks GB-2 and GB-9 started to lighten (Figure 8).

In all, three types of the gel pen inks, seven types of the fountain pen inks and all the erasable gel inks either became lighter or disappeared completely under irradiation at the different wavelengths, which contrasted with the results for the black photosensitive stamp-pad inks. Under the same conditions, all the black photosensitive stamp-pad inks remained unchanged. Therefore, a complete distinction between the black erasable gel inks and the black photosensitive stamp-pad inks could be made *via* infrared analysis, and three types of the black gel ink and seven brands of black fountain pen ink could be distinguished from the black photosensitive stamp-pad inks.

### Observable effects of fluorescence analysis

All the inks on printing paper were irradiated under the VSC6000’s built-in infrared luminescence (IRL) conditions. The 10 types of black photosensitive stamp-pad ink showed no fluorescence absorption (Supplement Figure S2).

**Figure 9.**
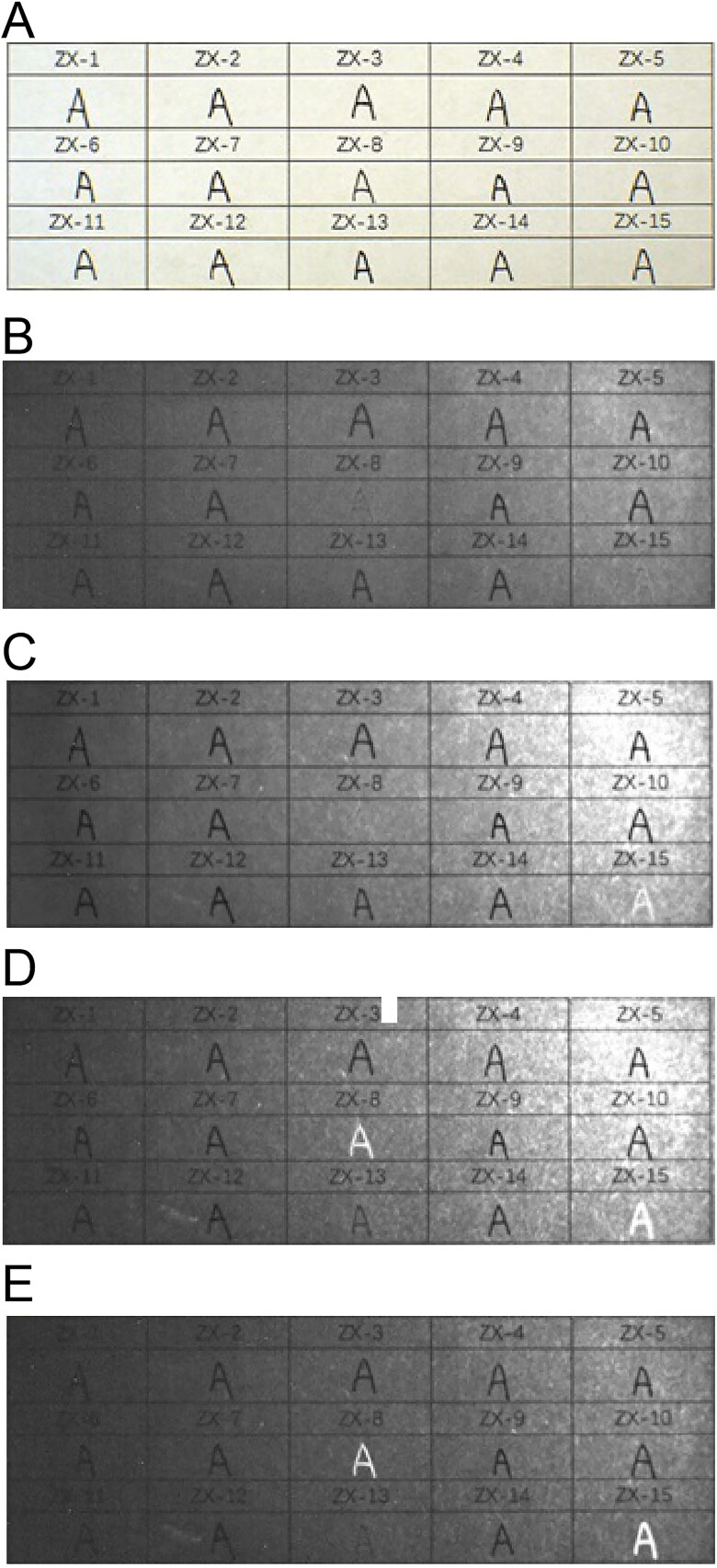
Changes in 15 types of black gel ink on printing paper with original diagram under visible light (A) and fluorescence absorption diagram under infrared luminescence (IRL) conditions with 400 to 485 nm spot filters and 645 nm long-pass filter (B), 445 to 570 nm spot filters and 645 nm long-pass filter (C), 515 to 640 nm spot filters and 695 nm long-pass filter (D), and 545 to 675 nm spot filters and 725 nm long-pass filter (E).

Among the 15 types of black gel ink, inks ZX-8 and ZX-15 exhibited weak fluorescence under the IRL conditions with the 400 to 485 nm spot filters and the 645 nm long-pass filter. Inks ZX-8 and ZX-15 were the brightest under the IRL conditions with the 515 to 640 nm spot filters and the 695 nm long-pass filter. The other gel inks showed no fluorescence absorption (Figure 9).

**Figure 10.**
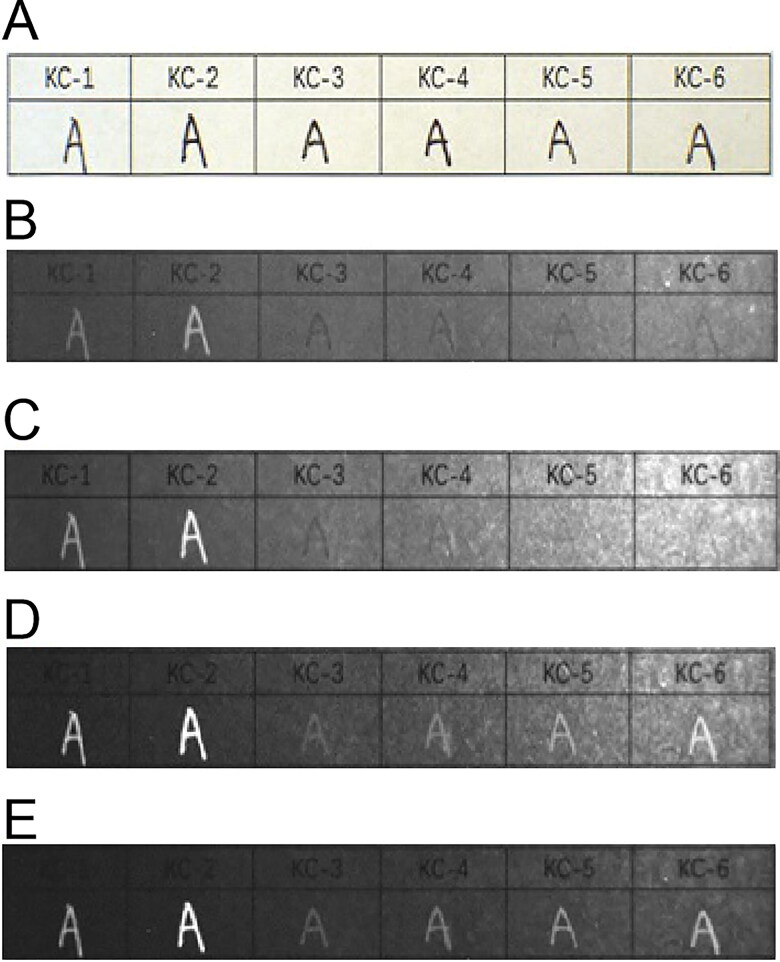
Changes in six types of black erasable gel ink on printing paper with original diagram under visible light (A) and fluorescence absorption diagram under infrared luminescence (IRL) conditions with 400 to 485 nm spot filters and 645 nm long-pass filter (B), 445 to 570 nm spot filters and 645 nm long-pass filter (C), 515 to 640 nm spot filters and 695 nm long-pass filter (D), and 545 to 675 nm spot filters and 725 nm long-pass filter (E).

As for the six types of erasable gel inks, inks KC-1 and KC-2 exhibited fluorescence under the IRL conditions with the 400 to 485 nm spot filters and the 645 nm long-pass filter. Under the IRL conditions with the 515 to 640 nm spot filters and the 695 nm long-pass filter, inks KC-3, KC-4, KC-5 and KC-6 also exhibited fluorescence in addition to inks KC-1 and KC-2. All the black erasable gel inks exhibited fluorescence and reached their brightest fluorescence state under the IRL conditions with the 545 to 675 nm spot filters and the 725 nm long-pass filter (Figure 10).

**Figure 11.**
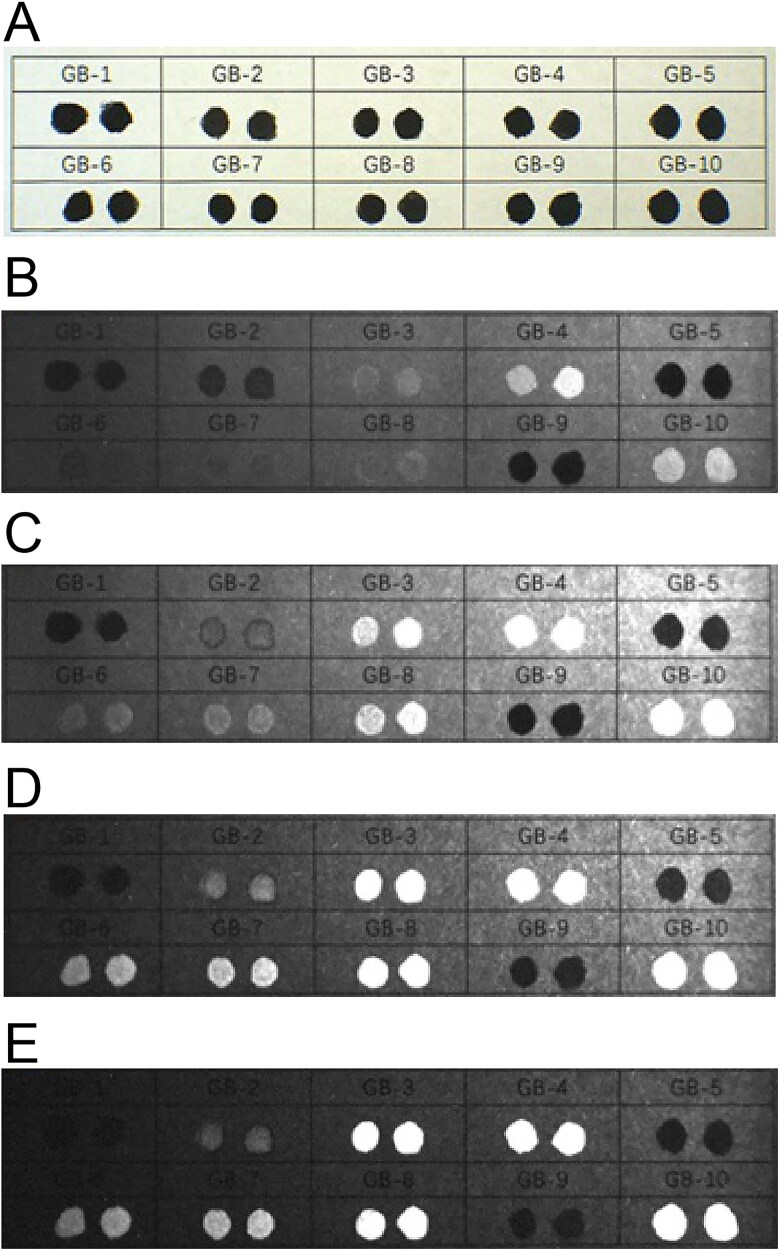
Changes in 10 types of black fountain pen ink on printing paper with original diagram under visible light (A) and fluorescence absorption diagram under infrared luminescence (IRL) conditions with 400 to 485 nm spot filters and 645 nm long-pass filter (B), 445 to 570 nm spot filters and 645 nm long-pass filter (C), 515 to 640 nm spot filters and 695 nm long-pass filter (D), and 545 to 675 nm spot filters and 725 nm long-pass filter (E).

With regard to the 10 types of fountain pen inks, under the IRL conditions with the 400 to 485 nm spot filters and the 645 nm long-pass filter, inks GB-3, GB-4, GB-7, GB-8 and GB-10 exhibited fluorescence but the other fountain pen inks showed no fluorescence absorption. Under the IRL conditions with the 445 to 570 nm spot filters and the 645 nm long-pass filter, inks GB-2 and GB-6 started to exhibit fluorescence; inks GB-3, GB-4, GB-7, GB-8 and GB-10 also showed brighter fluorescence than in the previous case. Under the IRL conditions with the 515 to 640 nm spot filters and the 695 nm long-pass filter, inks GB-2, GB-6 and GB-7 were brighter than in the previous case. Inks GB-3, GB-4, GB-8 and GB-10 also reached their brightest state of fluorescence in this case. Inks GB-2, GB-3, GB-4, GB-6, GB-7, GB-8 and GB-10 were the brightest under the IRL conditions with the 545 to 675 nm spot filters and the 725 nm long-pass filter. Inks GB-1, GB-5 and GB-9 showed no fluorescence absorption (Figure 11).

Overall, two types of gel pen ink, seven types of fountain pen ink and all the erasable gel inks exhi­bited fluorescence under irradiation at the different wavelengths, which differed from the results for the black photosensitive stamp-pad inks. Under the same conditions, all the black photosensitive stamp-pad inks had no fluorescence absorption. Therefore, a complete distinction between the black erasable gel inks and the black photosensitive stamp-pad inks could be made using fluorescence analysis, while only two types of black gel ink and seven types of black fountain pen ink could be distinguished from the black photosensitive stamp-pad inks.

### Observable effects of microspectrophotometry

**Figure 12.**
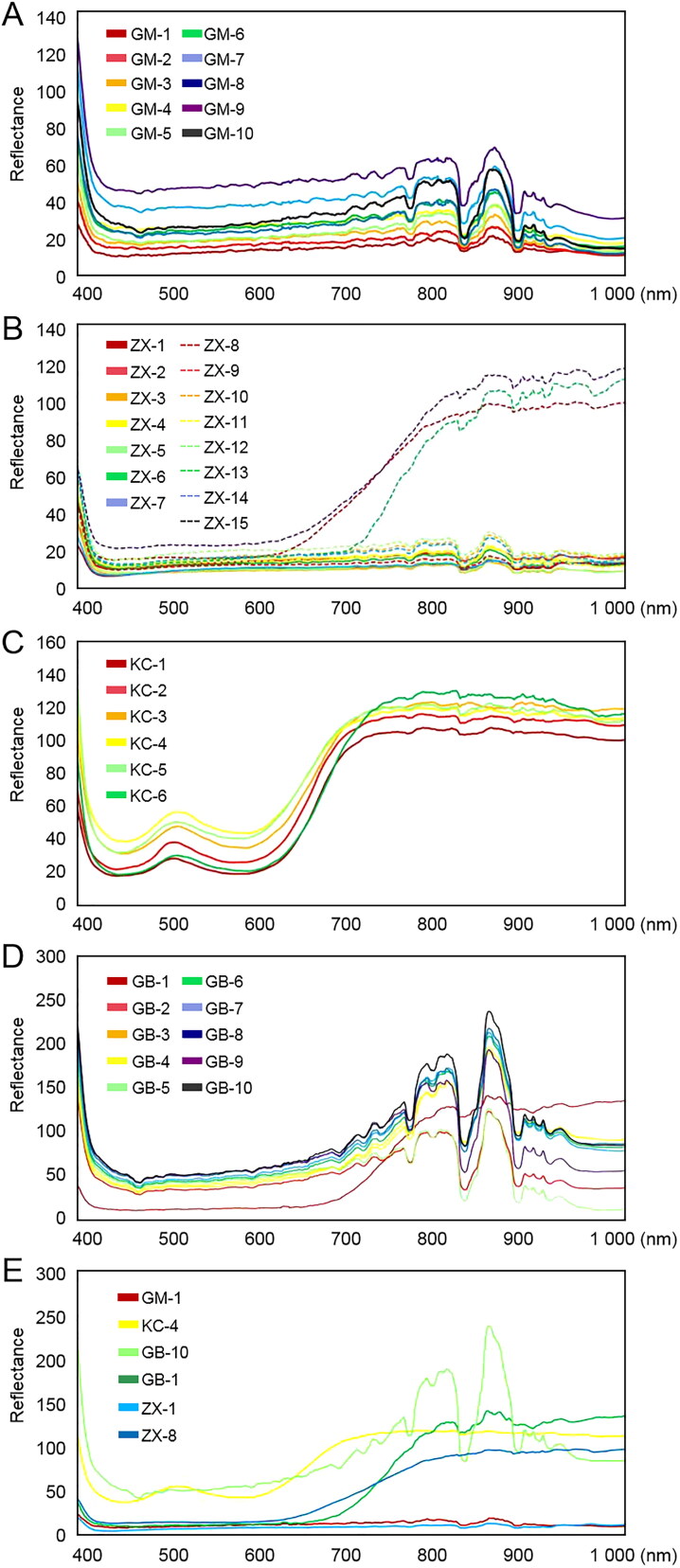
Microspectrophotometry spectra of 10 types of black photosensitive stamp-pad ink (A), 15 types of black gel ink (B), six types of black erasable gel ink (C), 10 brands of black fountain pen ink (D), and six types of representative ink (E) on printing paper.

All the inks were studied by microspectrophotome­try analysis and the spectra were obtained Figure 12. The spectra of the 10 types of black photosensitive stamp-pad ink were the same and there were no obvious features that could distinguish the individual inks (Figure 12A). Therefore, the spectra of these 10 black photosensitive inks could be represented using only one trend.

In the 15 types of black gel ink, with the exception of inks ZX-8, ZX-13 and ZX-15, the spectra of the ink samples were identical and there were no obvious features that could be used to distinguish these 12 types of gel ink (Figure 12B). Therefore, the spectra of the 15 black gel inks could be represented using two types of trend.

The spectra of the six types of black erasable gel ink were identical and there were no obvious features that could distinguish the six types of erasable gel ink (Figure 12C). Therefore, the spectra of the six black gel inks could be represented using only one trend.

As for the 10 types of black fountion pen ink, with the exception of ink GB-1, the spectra of the other samples were identical and there were no obvious features that could distinguish the nine types of fountain pen ink (Figure 12D). Therefore, the spectra of the 10 black fountain pen inks could be represented by two types of curve.

The spectra of the 10 types of black photosensitive stamp-pad ink, 15 types of black gel ink, six types of black erasable gel ink and 10 brands of black fountain pen ink were then compared *via* microspectrophotometry. To enable a more intuitive comparison, representative ink samples were selected to provide standard spectra for comparison: (1) black photosensitive stamp-pad ink: GM-1; (2) black gel ink: ZX-1 and ZX-8; (3) black erasable gel ink: KC-4; (4) black fountain pen ink: GB-1 and GB-10.

As shown in Figure 12E, ink GM-1, which provided the standard spectrum for the black photosensitive stamp-pad inks, showed a stable trend in the 400–1 000 nm range and no reflectance peak was observed. The overall trends of ink ZX-1, were simi­lar to those of GM-1 and could not be distinguished. Inks GB-1 and GB-10, showed an overall upward trend in the 700–800 nm range and a very obvious reflectance peak around the 800–850 nm region; ink KC-4 produced a reflectance peak near 500 nm and began to show an upward trend at 600 nm, which differed completely from the results obtained for GM-1; ink ZX-8 showed an overall upward trend near 650 nm. GB-1, GB-10, KC-4 and ZX-8 all differed completely from the results obtained for GM-1.

Therefore, the black photosensitive stamp-pad inks could be distinguished completely from all black fountain pen inks and all erasable gel inks using microspectrophotometry, but only three types of black gel ink could be distinguished from the black photosensitive stamp-pad inks using this method.

### Data analysis

All the inks were used in the MSP experiments. Each sample was tested six times resulting a total of 246 tests. The average value of each sample was taken to obtain the required spectrum.

To verify the reproducibility of MSP measurement, the reflectance peaks of 31 types of pen inks were tested for their significance. Ink GM-10 was selected randomly for 25 parallel tests. The five reflectance peaks with wavelengths of 726.14, 756.74, 795.37, 852.20 and 900.55 nm, were selected as the research objects. The six reflectance peak values of each peak were used as the test group. Twenty-five reflectance peak values of each peak that were measured in 25 parallel tests were recorded and were used as the control group (Supplementary Table S5). The two sets of data were then used for independent-sample T testing using IBM SPSS Statistics 23.0 software (Armonk, NY, USA) (Table 1). The results show that there is no signi­ficant difference between the means of the two sets of data. Therefore, the MSP spectrum of each ink shows good reproducibility.

In this study, principal component analysis (PCA) was used to process the MSP spectra of all four ink types. A PCA programme was developed using Matlab 2016 b software (MathWorks, Inc., Natick, MA, USA).

**Figure 13.**
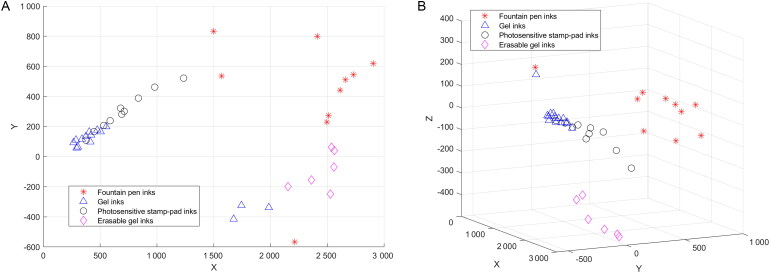
Principal component analysis (PCA) results for all MSP spectra. (A) PCA results based on two-dimensional vectors. (B) PCA results based on three-dimensional vectors.

Observation and analysis of the spatial distributions of the characteristic points representing the different inks shows that the characteristic points of the different ink types are located far apart in space, but the characteristic points of the same ink types are located relatively close together in space (Figure 13). Therefore, it can be demonstrated that the spectra (spectral data) are effective in enabling the different inks to be distinguished *via* PCA.

### Limitations of the analytical methods

The study mainly used optical methods to distinguish the different types and brands of the black gel ink, erasable gel ink and fountain pen ink from the photosensitive stamp-pad inks which reflect a certain objectivity. The experimental results demonstrate that the optical methods provide feasible ways to distinguish the other different types and brands of inks from the photosensitive stamp-pad inks, but there are still certain limitations to this approach. In other words, it is not possible to analyse the composition of all the inks qualitatively and quantitatively, as would be done in chemical testing. Therefore, it is impossible to distinguish the four inks above from their compositions alone. The ink composition analysis performed in this experiment is based on nondestructive examination. The results of the multiple ink analysis methods used above were summarized and the results are presented in Table 2. The ink composition analysis alone cannot necessarily distinguish the black photosensitive stamp-pad inks from the black fountain pen inks, gel inks, and erasable inks. After the IR, fluorescence and MSP analyses, it was found that the IR and fluorescence analyses and MSP could distinguish the black photosensitive stamp-pad inks from the fountain pen inks and the erasable gel inks very clearly, but only a few types of black gel ink could be distinguished from the black photosensitive stamp-pad inks. Therefore, ink composition analysis based on a nondestructive examination approach only would result in lower accuracy and lower detection rates. Assessment of other possible ink composition ana­lysis methods based on destructive examination, e.g. gas chromatography and mass spectrometry, will be our next research direction.

**Table 2.** Experimental results of different ink composition analysis methods to distinguish photosensitive stamp impressions and handwritten signatures.

Ink classification	Infrared analysis	Fluorescence analysis	Microspectro­photometry
Black fountain pen ink			
GB-1	√	χ	√
GB-2	χ	√	√
GB-3	√	√	√
GB-4	χ	√	√
GB-5	χ	χ	√
GB-6	√	√	√
GB-7	√	√	√
GB-8	√	√	√
GB-9	χ	χ	√
GB-10	√	√	√
Black gel ink			
ZX-1	χ	χ	χ
ZX-2	χ	χ	χ
ZX-3	χ	χ	χ
ZX-4	χ	χ	χ
ZX-5	χ	χ	χ
ZX-6	χ	χ	χ
ZX-7	χ	χ	χ
ZX-8	√	√	√
ZX-9	χ	χ	χ
ZX-10	χ	χ	χ
ZX-11	χ	χ	χ
ZX-12	χ	χ	χ
ZX-13	√	χ	√
ZX-14	χ	χ	χ
ZX-15	√	√	√
Black erasable gel ink			
KC-1	√	√	√
KC-2	√	√	√
KC-3	√	√	√
KC-4	√	√	√
KC-5	√	√	√
KC-6	√	√	√

“√” denotes that the analysis method can distinguish photosensitive stamp impressions from handwritten signatures and “χ” denotes that the analysis method cannot distinguish photosensitive stamp impressions from handwritten signatures.

In most cases, microscopic analysis could be used to determine whether a signature was handwritten or an impression made using a photosensitive signature stamp. The ink composition analysis approach is based on the premise that the microscopic analysis cannot determine clearly whether the signature was handwritten or an impression made using a photosensitive signature stamp, or is used to provide a clearer confirmation of the conclusions of the microscopic analysis from a quantitative perspective. The ink composition analysis represents a powerful supplementary approach to the microscopic analysis. A comprehensive examination method that includes microscopic analysis and ink composition analysis could therefore improve the accuracy rate of these examinations. While all experiments performed in this study are based on known black photosensitive stamp-pad inks, it is also recognized that it will be necessary to distinguish black photosensitive stamp-pad inks and gel inks from the chemical compositions of these inks. We will carry out further related research in this direction.

### Influence of the type of paper

This study used printing papers, writing papers and invoice papers to perform the microscopic analyses, infrared and fluorescence analyses, and microspectrophotometry analyses. The experimental results showed that there were no significant differences among the results of the IR and fluorescence analyses of the gel inks, fountain pen inks and erasable gel inks on the three white paper carriers listed above. Although the different types of white paper carrier had a certain impact on the experimental results, the black inks absorbed most of the incident light. Therefore, the specific white paper carrier had little effect on the experimental results obtained. With regard to experimental research on use of coloured paper as the carrier, because the proportion of coloured paper usage in actual cases was relatively small, no experiments or analyses were performed on coloured paper at this time. However, this does not rule out the possible effects of coloured paper on the experimental results. The premise of use of microspectrophotometry is to remove the paper background and thus exclude the influence of the paper from the measurement results. The experimental results obtained were based on single inks and thus changes in the type of paper carrier used in the microspectrophotometry procedure have no effect on the experimental results.

### Influence of applied pressure, temperature and the changes in the ink with time

Because the experiments were carried out in the same environment and under the same conditions, the possibility that the experimental results change under the condition where the ink is applied unevenly because of differences in the applied pressure is not excluded. The experimental results are also influenced by temperature and the paper used. Changes occurring in the ink over time may also interfere with the experimental results. Therefore, further experiments are required for validation and analysis of these aspects.

### Blind testing

Five laypersons and five FDEs participated in the blind testing process. Each layperson selected a gel pen, an erasable gel pen and a fountain pen at random to write their signatures, providing a total of 15 personal handwritten signatures. A signature written using a black gel pen was selected from each person to be used to fabricate a photosensitive signature stamp. Fifteen impressions from the photosensitive signature stamps and 15 signatures handwritten by the five laypersons were used as the questioned signatures. Signatures written using the black gel pens, black erasable gel pens and black fountain pens by each layperson were selected as specimen signatures. Each FDE then needed to examine the 30 questioned signatures and determine whether each of the signatures were handwritten or impressions made using the photosensitive signature stamps. A total of 150 blind tests were conducted. In addition, the FDEs were provided with training with regard to examination criteria and procedures to determine whether a questioned signature was handwritten or an impression made by the photosensitive signature stamp. After using microscopic analysis only, the results showed that the FDEs reached 142 correct conclusions and three qualified conclusions, with five inconclusive results, indicating that these FDEs achieved a 94.7% absolute accuracy rate and a 96.7% detection rate. The main reason for the qualified and inconclusive conclusions was that two of the questioned signatures were found to have no “squeegee effect”, scatter inks, ink defects or border features after microscopic ana­lysis. However, in these two signatures, only a few strokes showed writing indentations and these writing indentations were relatively shallow. Therefore, the opinions of the five FDEs were inconsistent. IR and fluorescence analyses and MSP were then used to examine the two questioned signatures by all five FDEs. For one of the two questioned signatures, four FDEs finally determined that the questioned signature was handwritten with a black erasable gel pen and gave a definite opinion, while the remaining FDE gave a qualified opinion. In the other questioned signature, one FDE finally could not determine whether it was a photosensitive signature stamp impression or handwritten with a black gel pen, three FDEs gave a qualified opinion and only one FDE always gave a definite opinion. The results after microscopic analysis and ink composition analysis showed that the five FDEs reached 145 correct conclusions, four qualified conclusions, and one inconclusive result, indicating that the FDEs achieved a 96.7% absolute accuracy rate and 99.3% detection rate. The blind test results presented in Table 3 demonstrate that the combination of these four analysis methods provides a more scientific approach to determine accurately whether a signature in question was handwritten or is a photosensitive signature stamp impression.

**Table 3.** Blind testing results.

Method	Correct conclusions	Qualified conclusions	No conclusion	Absolute accuracy rate (%)	Detection rate (%)
Microscopic analysis	142	3	5	94.7	96.7
Ink composition analysis	60	2	88	40.0	41.3
Microscopic and ink composition analysis	145	4	1	96.7	99.3

## Conclusion

Microscopic analysis, IR and fluorescence analyses and MSP were used to examine whether questioned signatures were handwritten or impressions made using a photosensitive signature stamp. In this study, 10 types of black photosensitive stamp-pad ink, 10 brands of fountain pen ink, 15 types of black gel ink and six types of black erasable gel ink were collected from the domestic market. In addition, 10 photosensitive signature stamps were fabricated using signatures from 10 people. Microscopic analysis, infrared and fluorescence analyses and MSP were used to examine the photosensitive signature stamp impressions when applied to printing papers, writing papers and invoice papers. The experimental results obtained reflect a certain objectivity. The results showed that microscopic analysis could distinguish between the photosensitive signature impressions and handwritten signatures in most cases. By comparing the optical characteristics of the photosensitive signature stamp impressions with those of signatures written using fountain pen inks, gel inks and erasable gel inks, it was found that infrared and fluorescence analyses and MSP could all distinguish the black photosensitive stamp-pad inks from fountain pen inks and erasable gel inks very clearly, but only three types of black gel inks could be distinguished from the black photosensitive stamp-pad inks. If the four optical analysis methods above are combined, they can provide a more scientific approach to determine whether a signature in question was handwritten or a photosensitive signature stamp impression. From 150 blind tests performed by five FDEs, it was demonstrated that a combination of these four analysis methods could determine whether the signature in question was handwritten or a photosensitive signature stamp impression effectively, with an absolute accuracy rate of 96.7% and detection rate of 99.3% being obtained.

It should be emphasized here that the results obtained from our experiments are only preliminary because of limitations of the types of pens and papers used and the time span available. Therefore, it will be necessary to carry out further tests in this area of study, including studies of the composition differences between photosensitive and gel inks, assessment of the validation criteria and further measurements on coloured paper.

## Authors’ contributions

Zhen Li drafted the manuscript. Xinlai Liu conceived the study.
